# A novel redox-responsive ursolic acid polymeric prodrug delivery system for osteosarcoma therapy

**DOI:** 10.1080/10717544.2020.1870583

**Published:** 2021-01-13

**Authors:** Daijie Fu, Zhe Ni, Kerong Wu, Peng Cheng, Xiaofeng Ji, Guoyuan Li, Xifu Shang

**Affiliations:** Department of Orthopedics, The First Affiliated Hospital of University of Science and Technology of China, Hefei, China

**Keywords:** Ursolic acid, redox-responsive, pH-responsive, polymeric prodrug, micelles, osteosarcoma

## Abstract

Ursolic acid (UA), found widely in nature, exerts effective anti-tumoral activity against various malignant tumors. However, the low water solubility and poor bioavailability of UA have greatly hindered its translation to the clinic. To overcome these drawbacks, a simple redox-sensitive UA polymeric prodrug was synthesized by conjugating UA to polyethylene glycol using a disulfide bond. This formulation can self-assemble into micelles (U-SS-M) in aqueous solutions to produce small size micelles (∼62.5 nm in diameter) with high drug loading efficiency (∼16.7%) that exhibit pH and reduction dual-sensitivity. The cell and animal studies performed using the osteosarcoma MG-63 cell line and MG-63 cancer xenograft mice as the model systems consistently confirmed that the U-SS-M formulation could significantly prolong the circulation in blood and favor accumulation in tumor tissue. Targeted accumulation allows the U-SS-M to be effectively internalized by cancer cells, where the rapid release of UA is favored by a glutathione-rich and acidic intracellular environment, and ultimately achieves potent antitumor efficacy.

## 1. Introduction

Osteosarcoma (OS) accounts for nearly 60% of all bone cancers (Duan et al., 2017). OS usually develops in the upper arm bone, shinbone, and thighbone. OS is often accompanied by a high risk of metastasis and death (Wu et al., 2016). Although chemotherapy has made significant progress to increase the survival rate of patients with the localized OS, the treatment of advanced, metastatic, and relapsed OS is still less than satisfactory, and the five-year survival rate of OS with metastases is only 20% (Wang et al., 2015; Zhang et al., 2016a). Additionally, severe side effects, such as myelosuppression, gastrointestinal toxicity, and hypersensitivity, and resistance toward conventional chemotherapy have greatly reduced the quality of life of patients (Shen et al., 2016; Zhang et al., 2017, 2018). Thus, there is an urgent need to identify superior and more efficacious treatment strategies.

Recently, traditional Chinese medicine, especially involving natural products, has attracted increasing attention as antitumor therapy. Ursolic acid (UA), a pentacyclic triterpenoid, can be isolated from various natural sources, such as *Salvia officinalis*, *Rosmarinus officinalis*, and *Sanguisorba officinalis* (Shao et al., 2020). It has been reported that UA has various pharmacological properties, including antitumor, anti-inflammatory, antibacterial, and antioxidant effects (Mlala et al., 2019; Shao et al., 2020). Previous studies have shown that UA can significantly inhibit proliferation and induce apoptosis of different types of malignant tumor cells, including those of breast cancer, hepatocarcinoma, and gastric cancer (Zhang et al., 2013, 2016a; Liu et al., 2017). Moreover, it is reported that UA can induce apoptosis by activating the ERK1/2 MAPK pathway and inactivating Wnt/β-catenin signaling in human OS cells (Wu et al., 2016; Zhang et al., 2016a). However, poor solubility, relatively short half-life, and low bioavailability have greatly limited the application of UA in the clinic (Baishya et al., 2016; Liu et al., 2017).

To increase the water solubility and bioavailability of UA, various nanotechnology-based drug delivery systems (DDSs) have been developed (Zhang et al., 2016b; Jiang et al., 2017; Ji et al., 2018; Liu et al., 2018; Poudel et al., 2020; Zhang et al., 2020a). These DDSs can be divided into two categories: noncovalent interactions and conjugation of UA to a polymer, according to the drug loading method. When compared with the entrapment of UA in nanocarriers, conjugation of UA to biocompatible polymers and the formation of polymer prodrugs displays various advantages, including an easy load of different drugs, facilitated modulation of drug loading content, prevention of premature release in the blood circulation, and controlled-drug release in response to endogenous and exogenous stimuli (Seidi et al., 2018; Yin et al., 2019). Most UA-polymer prodrugs have been synthesized by conjugating UA to the polymer through amide bonds or ester bonds, which then release UA under acidic conditions (Zhang et al., 2016b; Liu et al., 2017; Ji et al., 2018; Shen et al., 2018). However, the amide and ester bonds might be too stable inside cancer cells to allow the effective release of UA (Lv et al., 2014). Insufficient drug release may decrease antitumor efficiency of these UA prodrugs and may hinder their translation toward clinical applications (Chang et al., 2020). Therefore, a UA-prodrug that can effectively and rapidly release UA may significantly maximize the antitumor effects of UA.

It has been reported that the redox agent, glutathione (GSH), can achieve intracellular levels of 10 mM, while in the extracellular fluid its concentration is only about 2–20 μM (Guo et al., 2018; Chen et al., 2020; Zhang et al., 2020b). Moreover, the amount of GSH in cancer cells is fourfold higher than that of normal cells (Guo et al., 2018; Chen et al., 2020; Zhang et al., 2020b). Thus, GSH has been diffusely used as a perfect stimulus for controlled drug release (Raza et al., 2018). Disulfide bonds can be cleaved by GSH and this property has been widely used to develop redox-responsive DDS (Sun et al., 2018). For instance, Bao’s team described a GSH-responsive paclitaxel prodrug (TPGS-S-S-PTX), which was prepared by paclitaxel to TPGS through a disulfide bond (Bao et al., 2014). The MTT proliferation assay results showed that the TPGS-S-S-PTX formulation was 91% more effective than that of the redox-insensitive paclitaxel prodrug.

**Scheme 1.**
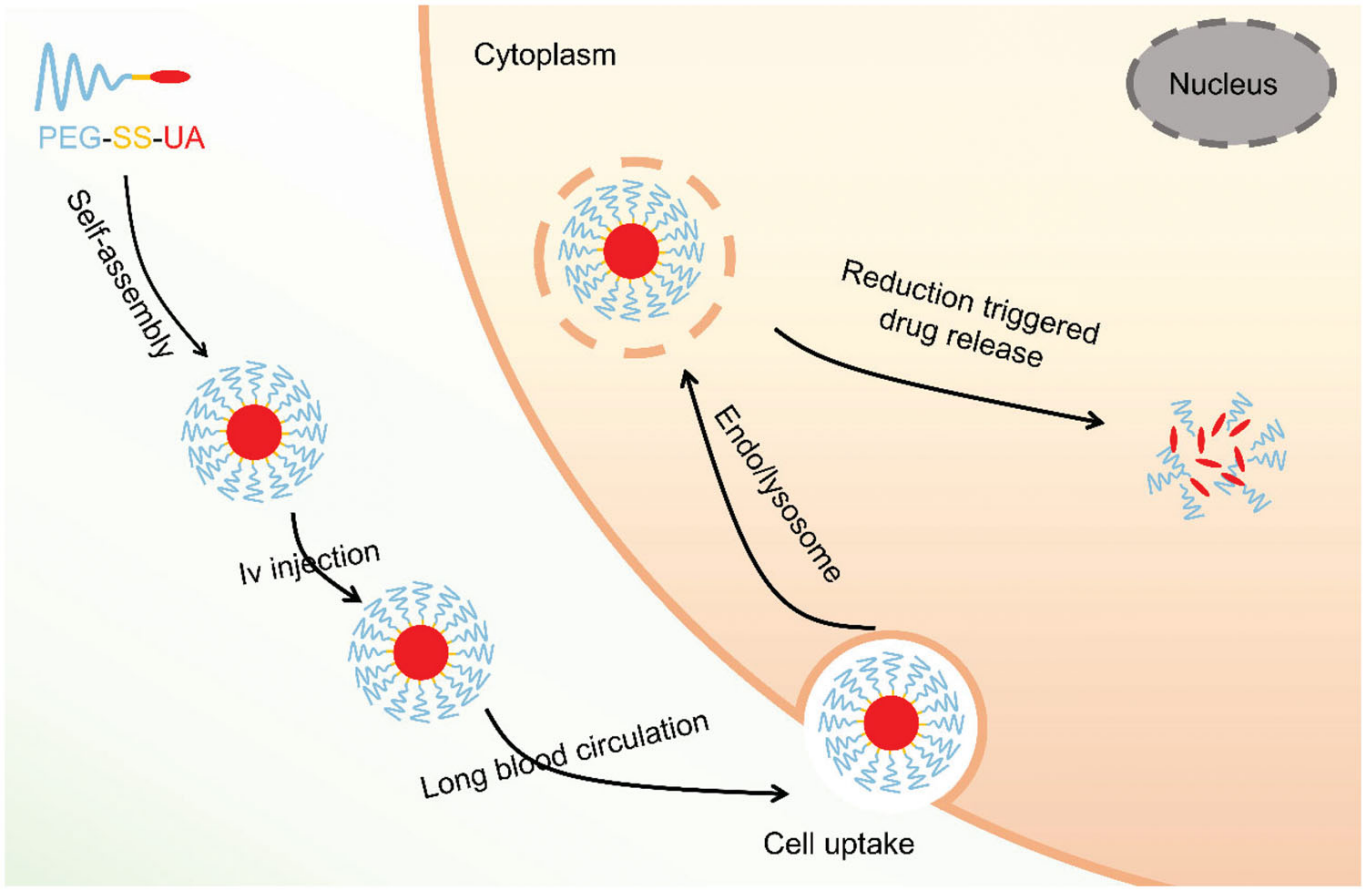
Preparation of U-SS-M formulation and GSH-responsive UA release in cancer cells.

Taking advantage of the significant differences between tumor tissue and normal tissue, we designed and prepared a redox-responsive UA polymeric prodrug, which was synthesized by conjugating UA to polyethylene glycol (PEG) through a disulfide bond (PEG-SS-UA). PEG-SS-UA can self-assemble to form a micelle (U-SS-M) in an aqueous solution (Scheme 1). U-SS-M can significantly increase the water solubility of UA, exhibit an excellent prolonged blood circulation time, and can selectively accumulate in tumor tissue *via* enhanced permeability and retention (EPR) effect (Yang et al., 2018; Goos et al., 2020) mediated-passive-targeting, and thus, sufficiently and selectively release the drug in tumor cells. U-SS-M can effectively suppress aggressive human OS MG-63 tumor growth *in vitro* and *in vivo*.

## 2. Materials and methods

### 2.1. Materials

Ursolic acid (UA), 1-ethyl-(3-dimethylaminopropyl) carbodiimide hydrochloride (EDC), succinic anhydride, 3,3′-dithiodipropionic acid (DTPA), and 4-dimethylamino pyridine (DMAP) were bought from Aldrich (Shanghai, China). Methoxypolyethylene glycol amine (PEG-NH_2_, molecular weight [MW]: 2000 Da) and methoxy PEG succinimidyl carboxymethyl ester (PEG-NHS, MW: 2000 Da) were obtained from Jenkem Technology Co., Ltd. (Beijing, China).

Human osteosarcoma MG-63 cells and mouse normal fibroblasts NIH-3T3 cells were brought from the Chinese Academy of Science Cell Bank for Type Culture Collection (Shanghai, China). MG-63 cells were maintained in a humidified incubator at 37 °C with 5% CO_2_ using MEM containing 10% fetal bovine serum (FBS, Every Green, Zhejiang Tianhang Biotechnology CO., LTD., China), 100 μg/mL streptomycin, and 100 IU/mL penicillin as the medium. NIH-3T3 cells were maintained in a humidified incubator at 37 °C with 5% CO_2_ using DMEM containing 10% fetal bovine serum (FBS, Every Green, Zhejiang Tianhang Biotechnology CO., LTD., China), 100 μg/mL streptomycin, and 100 IU/mL penicillin as the medium.

Sprague Dawley (SD) rat (male, 4–6 weeks old, 280–330 g) and BALB/c mice (male, 5–6 weeks, 18 ± 2 g) were purchased from Beijing Vital River Laboratory Animal Technology Co. Ltd and used under the approval of the Animal Care and Use Committee of the University of Science and Technology of China.

### 2.2. PEG-DTPA synthesis

The 3,3′-dithiodipropionic anhydride (DTDPA) was synthesized as a previous report (Jia et al., 2013). Briefly, 10.0 mL of acetyl chloride containing 2.0 g of DTPA was refluxed for 2 h at 65 °C, and then cooled to room temperature and concentrated to obtain the crude product. Subsequently, the crude product was precipitated in diethyl ether and washed with diethyl ether repeatedly, and then dried under vacuum to obtain DTDPA.

A solution consists of 1.0 g of PEG-NH_2_, 105.6 mg of DTDPA, and 40.0 mL of anhydrous *N,N*-dimethylformamide (DMF) was stirred for 10 h at 25 °C under an N_2_ environment. At the end of the reaction, the mixture was placed into a dialysis bag (molecular weight cutoff, MCW: 1000 Da) and dialyzed against DMF and water to remove any reacted molecules. Then, PEG-DTPA was obtained after lyophilization.

The structure of PEG-DTPA was assessed by ^1^H nuclear magnetic resonance (NMR) spectroscopy on a spectrometer operating at 300 MHz (Bruker AVANCE II, Switzerland).

### 2.3. PEG-SS-UA synthesis

To synthesize PEG-SS-UA, 80 mL of anhydrous DMF contained 27.3 mg of DMAP, 42.1 mg of EDC, 114.3 mg of UA, and 400.0 mg of PEG-DTPA were stirred under an N_2_ environment for 24 h at 25 °C. After the reaction, the mixture was dialyzed against DMF and water, and then lyophilized to obtain the PEG-SS-UA product.

For PEG-UA synthesis, PEG-NHS (400.0 mg) and UA (114.3 mg) were dissolved in 20 mL anhydrous DMF and stirred under an N_2_ environment for 24 h at 25 °C. After the reaction, the mixture was dialyzed against DMF and water, and then lyophilized to obtain the PEG-UA.

All products were confirmed by ^1^H NMR and high-performance liquid chromatography (HPLC) on a Shimadzu HPLC system (LC-20A, Japan) with the detector set at 282 nm using methanol: water: trifluoroacetate (80:20:0.05, v/v), as the mobile phase, using a C18 column (5 μm, 4.6 × 250 mm) (Agilent, USA).

### 2.4. Preparation of micelles

Micelles assembled by PEG-SS-UA (denoted UA-SS-M) and by PEG-UA (denoted UA-M) were synthesized via the simple solvent-evaporation method. Briefly, the preparation of U-SS-M will be described to illustrate the method. Typically, 30.0 mg of PEG-SS-UA was dissolved in 1.0 mL of ethanol under sonication. Subsequently, the solution was slowly added to 10.0 mL distilled water dropwise under continuous stirring. The ethanol was removed by stirring the micellar solution overnight. Finally, the mixture was filtered through a 0.4 μm filter to obtain the U-SS-M micelles.

The drug loading efficiency (DLE) of the prodrug micelles was measured by HPLC method as mention above and calculated using the following equation:

$$\mathrm{DLE}\;(\%\mathrm{wt})\;=\;\frac{\text{UA weight}}{\text{Micelles weight}}\;\times \;100\%$$

Coumarin-6 loaded U-SS-M and U-M micelles were also prepared using the same method. Briefly, 10.0 mg of PEG-SS-UA (in the alternative, PEG-UA) and 0.5 mg coumarin-6 were dissolved in 0.5 mL ethanol under sonication. Subsequently, the solution was slowly added to 3.0 mL of distilled water dropwise under continuous stirring. The ethanol was removed by stirring the micellar solution overnight. Finally, the mixture was filtered through a 0.4-μm filter to obtain the coumarin-6 loaded U-SS-M (or U-M) micelles.

The zeta potential, size, and polydispersity index (PDI) in an aqueous solution of these micelles were detected by dynamic light scatting (DLS) on a Malvern Zetasizer Nano-S90 (UK); and the structure and morphology of each micelle were recorded on a transmission electron microscope (TEM, JEOL JEM-1200EX microscope, Japan).

### 2.5. Evaluation of micelles stability

To explore the ability of the micelle to maintain stability under storage conditions and *in vivo* looping environment, micelles were incubated in PBS or PBS containing 10% FBS at 37 °C. At predetermined time intervals, the size of the micelles was recorded.

### 2.6. Evaluation of redox-sensitivity

To study the redox-sensitivity of the prepared micelles, size changes of the micelles and they are *in vitro* drug release properties under various redox-conditions were measured. To investigate the size changes of micelles, U-SS-M and U-M were incubated in PBS at pH 7.4, PBS at pH 5.0, and PBS at 7.4 containing 10 mM GSH for 2, 4, or 8 h at 37 °C, respectively, and the size and PDI of the micelles were detected by DLS.

The *in vitro* release experiment was performed in PBS at various conditions: pH 7.4, pH 7.4 with 20 μM GSH, pH 7.4 with 10 mM GSH, pH 5.0, and pH 5.0 with 10 mM GSH, respectively. Briefly, freshly prepared U-SS-M or U-M micelles (containing about 2.0 mg of UA) were suspended in 4.5 mL of release medium and then were placed into a dialysis bag (MWCO: 1000 Da). Subsequently, the dialysis bag was immersed in 45.5 mL of release medium and gently shaken in a thermotank at 37 °C. At the predetermined time point, 1.0 mL of the sample was removed from the incubation medium and replaced with an equal volume of fresh release medium. The amount of UA was determined HPLC method as described above.

### 2.7. Cellular uptake

Cell internalization of coumarin-6-loaded U-SS-M or U-M micelles were observed using a confocal laser scanning microscope (CLSM). MG-63 cells were seeded into a six-well plate with coverslip at a density of 1.0 × 10^5^ cells per well. After culturing overnight, the cells were treated with coumarin-6-loaded U-SS-M or U-M micelles for 1 or 3 h. After treatment, the cells were rinsed with PBS three times, fixed with formaldehyde (4% in PBS) for 10 min, and then stained with DAPI for 10 min, washed with PBS, and then observed under a CLSM (Carl Zeiss LSM 700, Germany). All steps were performed at room temperature.

### 2.8. *In vitro* cytotoxicity

MG-63 cells and NIH-3T3 cells were seeded in 96-well plates at a density of 5000 cells/well. After culturing overnight, the cells were treated with UA, U-SS-M, or U-M at various concentrations. After treatment for 48 h, 20.0 μL of MTT solution was added to each well and then the cells were cultured for a further 3 h. After incubation, the solution was replaced with 150.0 μL DMSO followed by shaking for 15 min, then the adsorption of the solution was measured using a microplate reader (Bio-Rad 680, USA) at 490 nm. The equation: (A_tretment_/A_control_) × 100, was used to calculated relative cell viabilities, where A_treatment_ and A_control_ represented the absorbances of the treatment well and control well, respectively.

### 2.9. Pharmacokinetics and biodistribution

The pharmacokinetics of the UA micelles were evaluated using SD rats as the animal model. Rats were treated intravenously with UA, U-SS-M, or U-M (equal to free UA 11.0 mg/kg) *via* the tail vein. At predetermined time intervals, a 500.0 μL volume of the blood sample was obtained from the orbital plexus. The samples were weighed and centrifuged immediately at 3000 rpm at 4 °C for 10.0 min. A 1.0 mL volume of ethyl acetate was added to the supernatant, which was then sonicated, and centrifuged. The organic solvent was collected, dried under vacuum, redissolved in 100 μL methanol, 50 μL 0.1 M HCl, then sonicated and centrifuged. The precipitate was redissolved using methanol and then analyzed by HPLC as described above.

The biodistribution of UA micelles was investigated using MG-63 tumor-bearing mice as the animal model. MG-63 cancer cells xenograft mice were prepared by the injection of six million cells into the left flank of BALB/c mice. Twenty days following implantation, mice were treated intravenously with UA, U-SS-M, or U-M (equal to free UA 11.0 mg/kg) *via* the tail vein. After a 6-h or 12-h treatment, six mice per treatment were sacrificed and tumors and organs (kidney, heart, lung, spleen, and liver) were collected, weighed, and pulverized. The subsequent steps were the same as those used for processing the pharmacokinetic samples.

### 2.10. *In vivo* antitumor efficiency

MG-63 tumor-bearing mice were allocated to five groups randomly. Until the tumor volume reached about 80 mm^3^, mice were intravenously injected with saline and 11.0 mg/kg UA equivalents of UA, U-SS-M, and U-M every three days for a total of five times. Mouse body weight and the tumor length and width were detected every three days after the first administration. The equation: tumor volume = length × (width)^2^/2, was used to calculate the tumor volume.

On day 21, all mice were sacrificed and collected from the tumor tissues. Subsequently, the tumor tissues were weighed and imaged. The degree of tumor growth inhibition (TGI) was calculated according the following equation:

$$\mathrm{TGI}\;(\%)=\frac{\left({\text{tumor weight}}_{\mathrm{saline}}\;–\;{\text{tumor weight}}_{\mathrm{treatment}}\right)}{{\text{tumor weight}}_{\mathrm{saline}}}\times \;100\%.$$

## 3. Results and discussion

### 3.1. The fabrication and characterization of UA polymeric prodrug

**Scheme 2.**
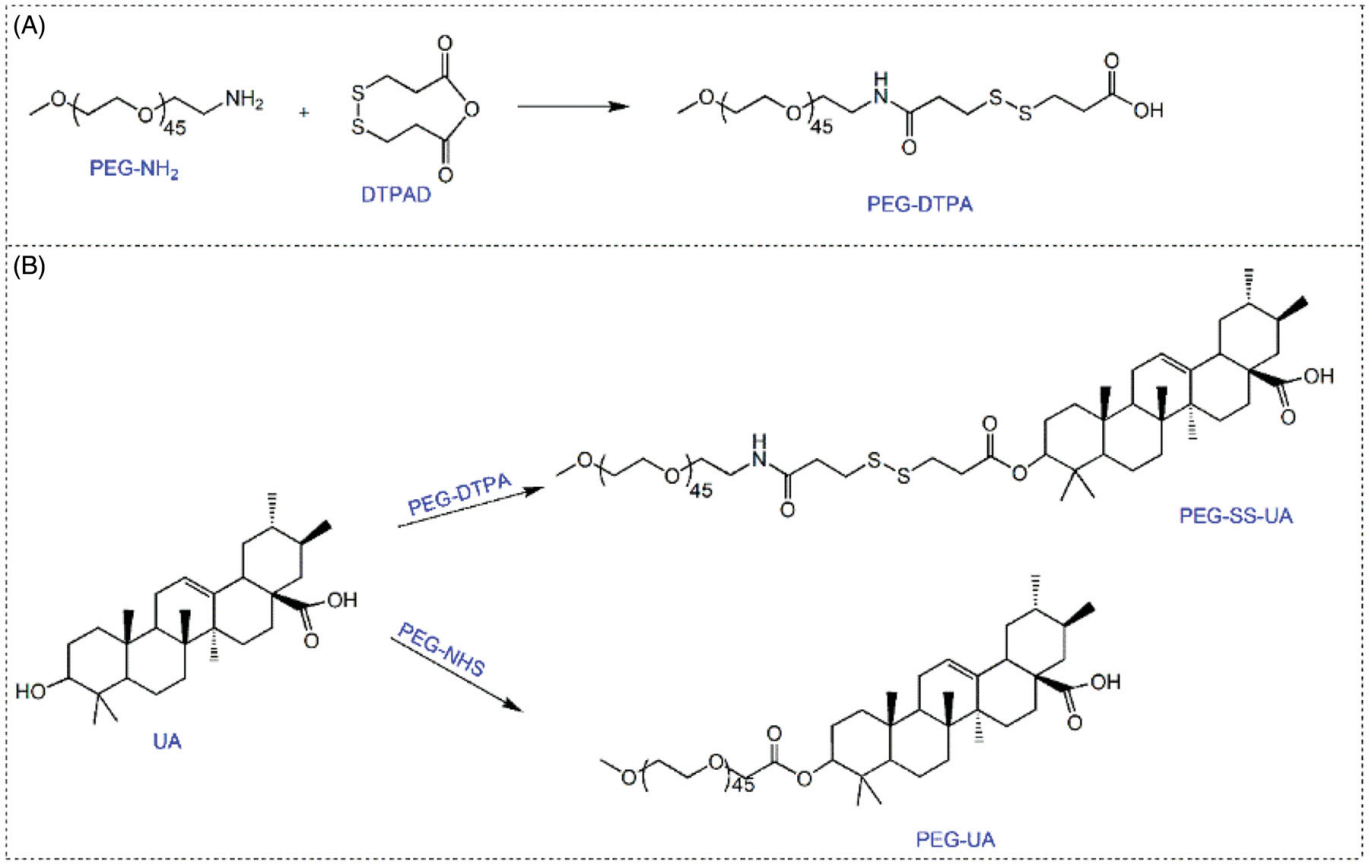
Synthesis of PEG-SS-UA and PEG-UA.

**Figure 1.**
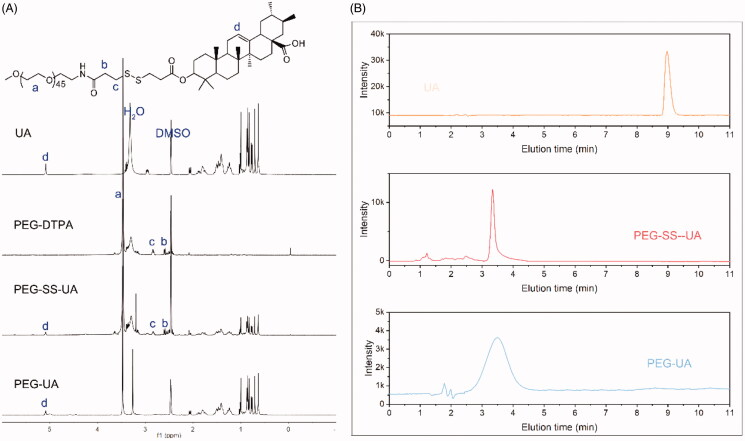
(A) ^1^H NMR spectra of UA, PEG-DTPA, PEG-SS-UA, and PEG-UA using DMSO-*d6* as the solvent. (B) HPLC spectra of UA, PEG-SS-UA, and PEG-UA.

Redox-sensitive and redox-insensitive UA polymeric prodrugs were synthesized *via* esterification, as illustrated in Scheme 2. To improve the reaction efficiency of PEG and DTPA, the DTDPA was synthesized first, as it exhibits high reactivity with the primary amino groups (Bao et al., 2014). Moreover, this reaction can also limit the production of byproduct PEG-DTPA-PEG. ^1^H NMR was employed to assess the configuration of all products. As shown in Figure 1, typical peaks of the two methylene groups of DTPA appeared at 2.6 and 2.8 ppm in the PEG-DTPA spectra, and the signal at 3.5 was the characteristic peak of PEG. These results demonstrated that DTPA was successfully conjugated to PEG. In the PEG-SS-UA spectra, the characteristic peaks at 5.3 belonged to the protons of the olefinic double bonds of UA, and signals at 0.8–2.2 ppm were attributed to the peaks of the pentacyclic triterpene of UA. Moreover, the typical peaks of PEG and DTPA also appeared in the PEG-SS-UA spectra, these results suggested that PEG-SS-UA was successfully prepared. Similarly, the characteristic peaks of PEG and UA could be observed in the ^1^H NMR spectra of PEG-UA, confirming that PEG-UA was also successfully produced. Moreover, we further evaluated the purity of PEG-SS-UA and PEG-UA using HPCL (Figure 1(B)). In both the HPLC spectrums of PEG-SS-UA and PEG-UA did not found the UA peak, indicating that the high purity of PEG-SS-UA and PEG-UA, as well as the successfully synthesized of both prodrugs.

### 3.2. Micellar preparation and characterization

**Figure 2.**
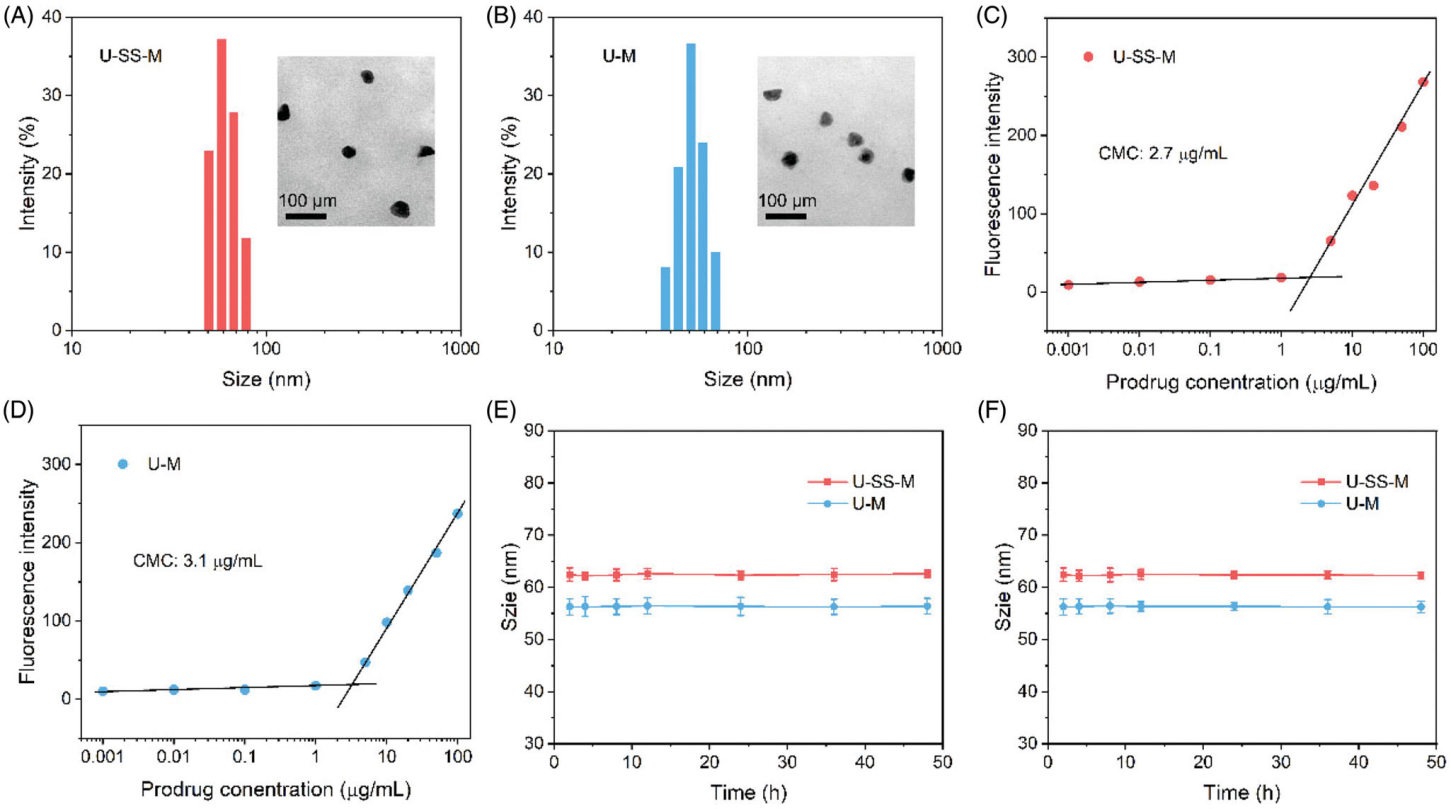
Characterization of micelles. (A, B) Size distribution and TEM images of U-SS-M (A) and U-M (B). (C, D) CMC values of U-SS-M (C) and U-M (D). (E, F) Size changes of U-SS-M and U-M micelles following incubation in PBS (E) and PBS supplemented with 10% FBS (F) for various times (*n* = 3).

The amphiphilic UA polymeric prodrug can self-assemble in an aqueous solution. The redox-sensitive micelles, assembled by PEG-SS-UA (U-SS-M) and the redox-insensitive micelles formed by PEG-UA (U-M) were subjected to TEM and DLS to assess morphology and size distribution. As shown in Figure 2(A,B), all micelles exhibited a uniform spherical shape. The size of U-SS-M and U-M micelles were 93.5 and 97.5 nm, respectively (Figure 2(A,B) and Table 1). The moderate particle size is conducive to the accumulation of these micelles in tumor tissues through the EPR effect (Chang et al., 2020). The zeta potentials of U-SS-M and U-M were −9.7 and −9.3 mV, respectively (Table 1). The negative charge was attributed to the PEG shell, which may reduce the interaction of nanoparticles with blood components, thus, the slightly negative zeta potential could contribute to these micelles having better stability in blood and increased blood circulation time (Lv et al., 2014). The DLE of U-SS-M and U-M detected by the HPLC method was 16.7 wt% and 17.3 wt%, respectively. This is higher than many traditional UA-based delivery systems (Zhang et al., 2013; Baishya et al., 2016).

**Table 1.** Characterization of U-SS-M and U-M micelles.

Micelles	Size (nm)	PDI	Zeta potential (mV)	DLE (wt%)
U-SS-M	62.5 ± 1.3	0.20 ± 0.02	−13.7 ± 1.6	16.7 ± 1.1
U-M	56.3 ± 1.6	0.22 ± 0.03	−14.1 ± 0.9	17.3 ± 0.8

The CMC values of U-SS-M and U-M were 2.7 and 3.1 μg/mL (Figure 2(C,D)), respectively, which were measured using Nile red as the fluorescence probe. The different CMC values may be due to the different linkers between PEG and UA (Bao et al., 2014). The small CMC values of both micellar preparations could protect them against dilution effects in the blood circulation, thereby improving their stability (Chen et al., 2017). Subsequently, the stability of these micelles in PBS and PBS supplemented with 10% FBS was investigated. As shown in Figure 2(E,F), the sizes of U-SS-M and U-M showed no significant changes within the 48 h incubation, suggesting these micelles could maintain stability in the blood circulation, ultimately improving drug delivery.

### 3.3. Evaluation UA micelles redox-sensitivity

**Figure 3.**
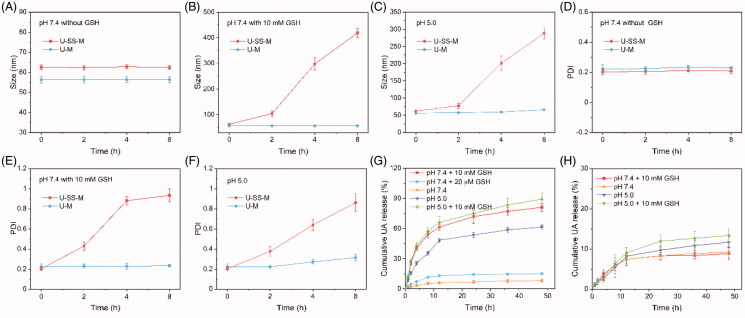
Redox-sensitivity studies. Size (A–C) and PDI (D–F) changes of U-SS-M and U-M at pH 7.4 with or without 10.0 mM GSH (*n* = 3). Cumulative release of UA from U-SS-M (G) or U-M (H) at pH 7.4 with 10.0 mM GSH, pH 7.4 with 20 μM GSH, pH 7.4, or pH 5.0, respectively (*n* = 3).

According to our hypothesis, the disulfide linkage between UA and PEG will be cleaved once the U-SS-M are internalized by cancer cells. To investigate their redox-responsive capacity, the size changes of U-SS-M and U-M under various redox conditions were measured. In addition, many previous reports showed that the β-thiopropionate linkage was not only cleaved by reductive stimuli but it could also be degraded by acidic conditions (Lv et al., 2014; Zou et al., 2014). It is well known that within normal tissue pH is ∼7.4, but within the lysosome and endosome the pH is ∼5–6 (Zou et al., 2014; Mu et al., 2019). Thereby, U-SS-M may degrade in cell lysosome and endosome acidic condition. To prove this hypothesis, size changes of U-SS-M and U-M at pH 5.0 was also evaluated. As shown in Figure 3(A–D), after incubation for 8 h, size and PDI values of the redox-insensitive U-M did not show any remarkable changes both in the high concentration of GSH and acidic conditions. Similarly, the U-SS-M also remained stable in the absence of GSH, as evidenced by the lack of a remarkable shift in size or the PDI (Figure 3(A,D)). However, the size and PDI of U-SS-M rapidly increased from 63 nm/0.20 to 419 nm/0.93 upon treatment with 10.0 mM GSH for 8 h (Figure 3(B,E)), indicating the high sensitivity of the micellar system to reducing conditions. Moreover, the size and size and PDI of U-SS-M were also significantly changed after cultured in an acidic condition (Figure 3(C,F)), suggesting the pH-responsive ability of U-SS-M. The underlying mechanism involved the removal of UA by GSH and acidic, which transformed the hydrophobic core to a hydrophilic one and then induced the disassembly of the U-SS-M. These results showed that the U-SS-M have GSH- and pH-dual responsive capability.

Subsequently, the redox-responsive drug release properties of the U-SS-M preparation were investigated by dialysis. As shown in Figure 3(G), in the physical and extracellular environment at pH 7.4 and pH 7.4 containing 20.0 μM GSH, negligible amounts of UA were released from U-SS-M (≤16%) within 48 h, further demonstrating the good stability of the U-SS-M. In contrast, upon incubation with 10.0 mM GSH for 48 h, over 80% of UA was released from the U-SS-M, confirming its redox-responsive drug release properties. To further confirm the pH-sensitive ability of U-SS-M, drug release properties of U-SS-M at pH 5.0 was also investigated. As shown in Figure 3(G), approximately 60% of UA was released from U-SS-M after a 48-h incubation, suggesting that pH-triggered UA release had occurred. Additionally, the U-SS-M release was further accelerated upon incubation at high GSH concentration and acidic combination conditions. These results further demonstrated the GSH and pH-dual responsive ability of U-SS-M. In comparison, only a small quantity of UA (below 9%) was released from U-M following incubation at pH 7.4 or under high GSH condition (pH 7.4 with 10.0 mM GSH, Figure 3(H)), demonstrating the reduction-insensitive properties of the U-M preparation. Moreover, when the pH decreased to 5.0, only ∼11.7% and ∼13.3% of UA were released from the U-M preparation after treated with or without 10.0 mM GSH (Figure 3(H)), respectively. The release behaviors of UA from the U-M preparation demonstrated that the UA polymeric prodrug synthesized by conjugating UA to the polymer by an ester or amino bond was highly stable at various extracellular and intracellular conditions, but may strongly inhibit the antitumor efficacy of UA in tumor tissues. In comparison with normal tissue, most solid tumors have higher redox status and acidic environments, thus, the U-SS-M micelles with redox and pH dual-sensitive drug release properties could effectively avoid premature cargo leakage in the blood, so as to significantly decrease the drug-related side effects and rapidly and specifically release the drug at the tumor-specific environments.

### 3.4. Cell uptake

**Figure 4.**
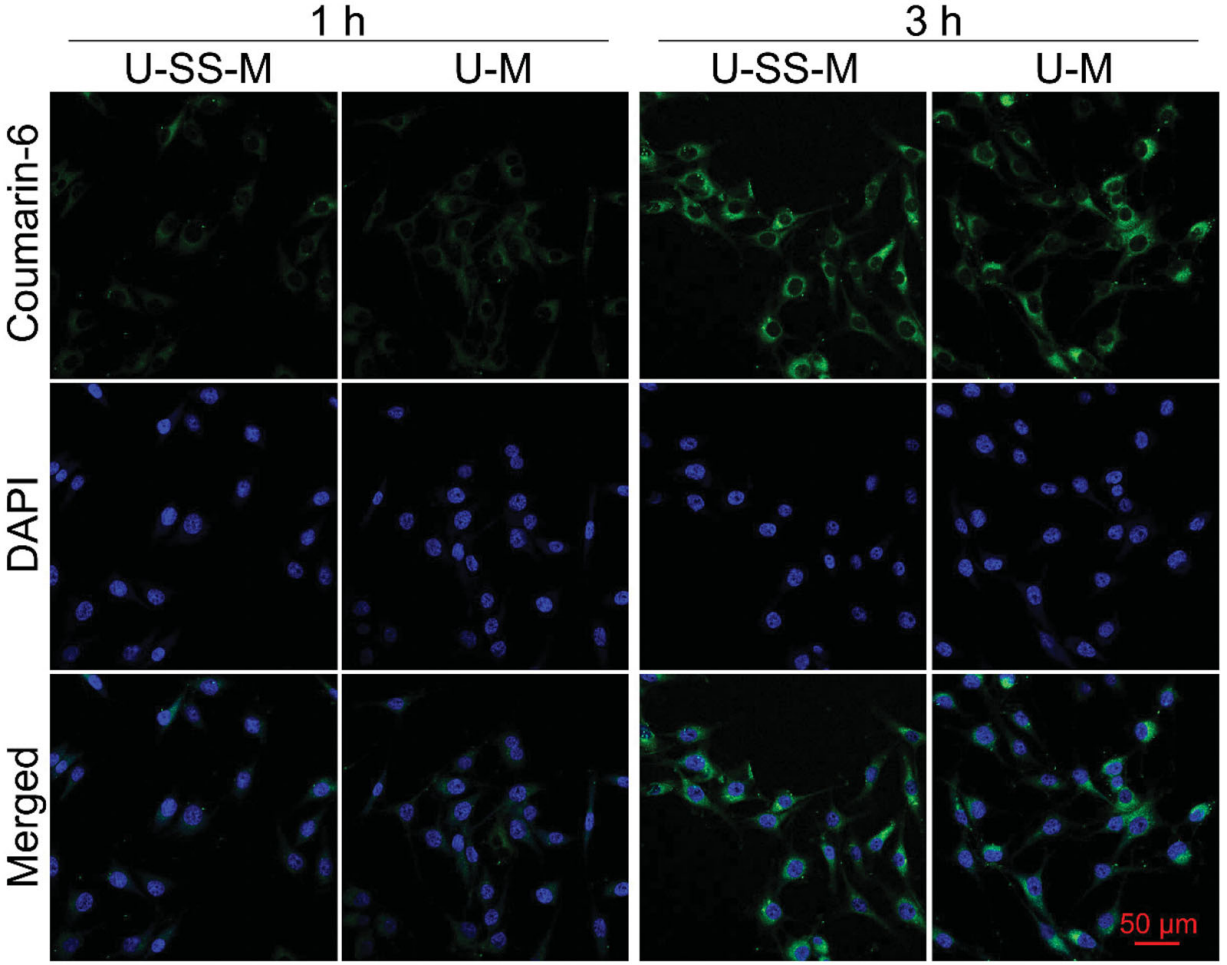
CLSM images of MG-63 cells treated with coumarin-6-loaded U-SS-M and U-M for 1 and 3 h, respectively.

The cellular uptake behavior of coumarin-6-loaded U-SS-M and U-M was studied in human OS MG-63 cells by CLSM. As exhibited in Figure 4, the cell internalization process of U-SS-M and U-M was similar and time-dependent. A weak green fluorescence signal of coumarin-6 can be found in the cytoplasm after 1 h treatment. After the treatment time was prolonged to 3 h, the green fluorescence intensity was increased both in the U-SS-M and U-M groups, indicating the cell uptake levels of these micelles were increased. These results demonstrated that the U-SS-M and U-M micelles could be successfully internalized by MG-63 cells.

### 3.5. *In vitro* cytotoxicity

**Figure 5.**
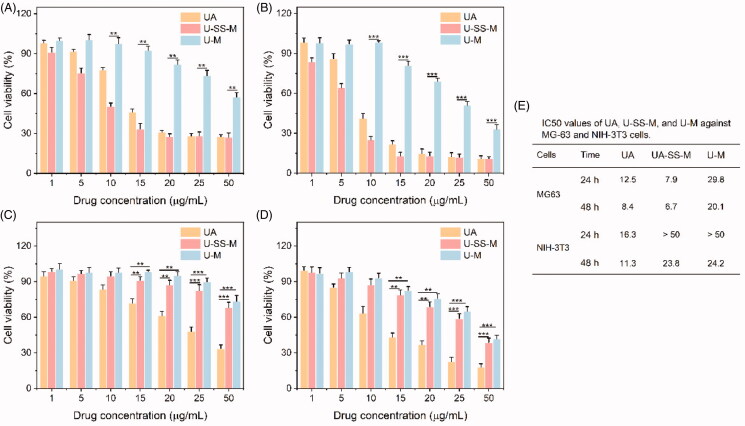
*In vitro* cytotoxicity. Cell viability of MG-63 cells after treatment with UA, U-SS-M, and U-M for 24 h (A) and 48 h (B) (*n* = 6). ** *p* <.01, *** *p* <.001. Cell viability of NIH-3T3 cells after treatment with UA, U-SS-M, and U-M for 24 h (C) and 48 h (D) (*n* = 6). ** *p* ≤.01, *** *p* ≤.001. (E) IC50 values of UA, U-SS-M, and U-M against MG-63 cells and NIH-3T3 cells.

To study the anti-OS effects of the redox-sensitive UA polymeric prodrug preparation, the *in vitro* cytotoxicity of UA, U-SS-M, and U-M against MG-63 cells after a 24 h or 48 h exposure was measured by the MTT method. As presented in (Figure 5(A,B,E)), all the UA formulations displayed a dose- and time-dependent growth inhibitory effect. The U-SS-M exhibited the best antitumor activity at both 24 h and 48 h. The IC50 at 24 h and 48 h was 7.9 and 6.7 ug/mL, which was 1.6-/3.8-fold and 1.3-/3.0-fold lower than that of free UA and U-M, respectively. Free UA showed worse cytotoxicity than U-SS-M, which was probably due to the lower solubility of the UA. As mentioned above, U-SS-M and U-M exhibited similar cellular uptake behavior, thus, the superior cancer proliferation inhibition properties of U-SS-M were mainly due to rapid and complete intracellular drug release.

Moreover, the cell proliferation inhibition properties of all drug formulations against normal NIH-3T3 cells were also evaluated using the MTT method to further investigate the redox-responsive drug release of U-SS-M (Figure 5(C–E)). Similarly, all the UA formulations exhibited a dose- and time-dependent growth inhibitory effect. In comparison with free UA, U-SS-M and U-M showed slightly cytotoxicity, which may be caused by intracellular incomplete drug release. These results further suggesting that the U-SS-M with redox and pH dual-responsive ability may have remarkably tumor selectively cytotoxicity.

### 3.6. Pharmacokinetics and biodistribution of the micellar preparation

**Figure 6.**
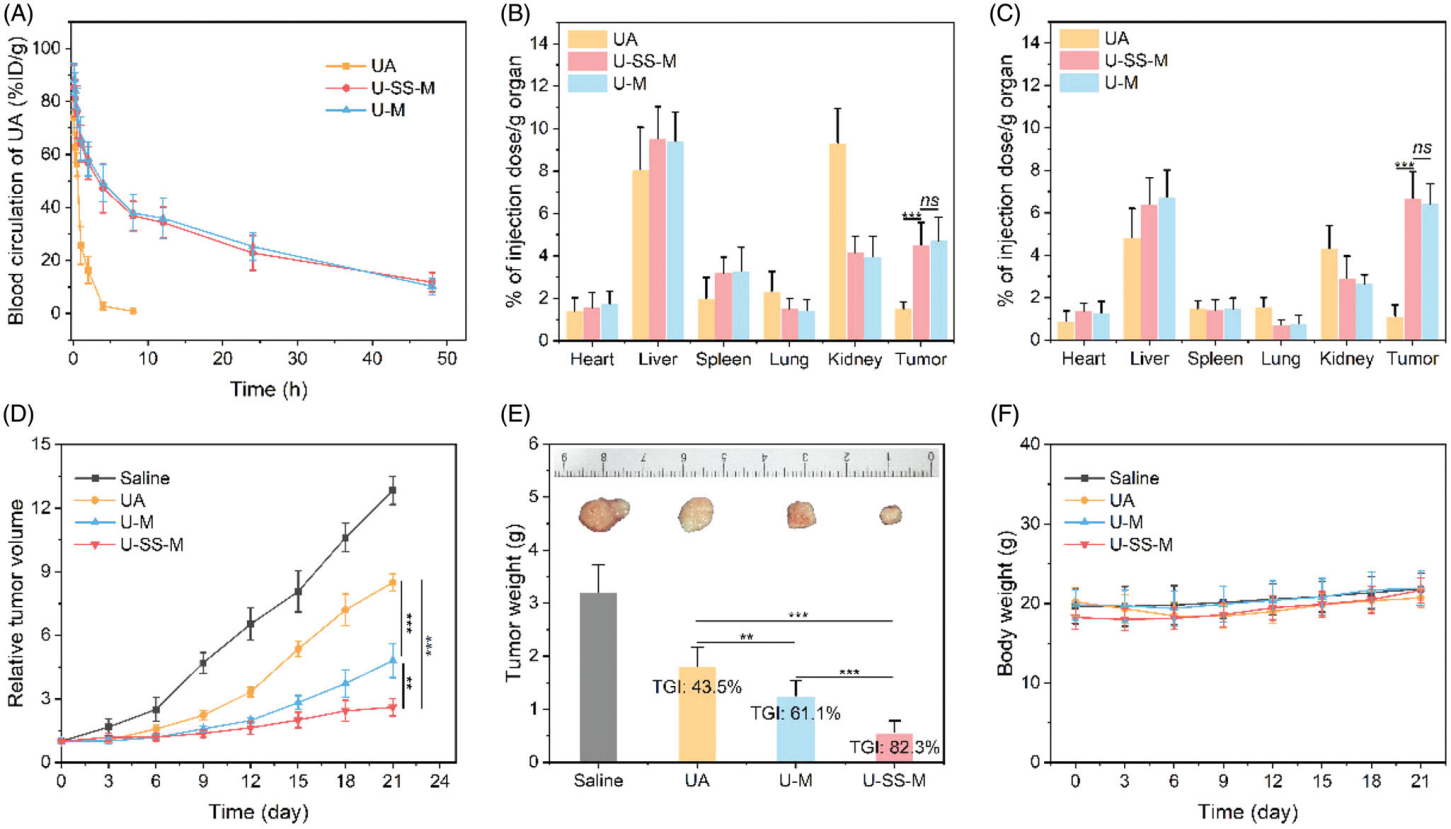
*In vivo* studies. (A) Blood levels of free UA, U-SS-M, and U-M in SD-rats (*n* = 6). Distribution of UA, U-SS-M, and U-M in the tumor tissue and organs (kidney, lung, spleen, liver, and heart) after injection at 6 h (B) and 12 h (C) in MG-63 tumor bearing mice (*n* = 6). *ns*: no significantly difference; *** *p* <.001 (D) Tumor volume changes after treated with saline, free UA, U-SS-M, or U-M (*n* = 6). ** *p* <.01, *** *p* <.001. (E) Tumor weight, tumor growth inhibition (TGI), and representative images of tumors at day 21 (*n* = 6). ** *p* <.01, *** *p* <.001. (F) Body weight changes of mice after treated with saline, free UA, U-SS-M, or U-M (*n* = 6).

The low water solubility of UA results in poor pharmacokinetics *in vivo*, which limits the effectiveness of UA (Zhang et al., 2013). Our UA polymeric prodrug product significantly increased the solubility of UA, which may contribute to improving its pharmacokinetics. The blood drug concentration changes were monitored to confirm these properties. As shown in Figure 6(A), the three UA formulations showed a biphasic clearance in normal SD rats. Free UA was rapidly cleared from the blood. Half of the injected dose of UA was eliminated within 10 min, and no drug could be detected in the blood after 10 h, which was consistent with the results of a previous report (Liu et al., 2017). In contrast, the UA in U-SS-M and U-M cleared relatively slowly and retained a high concentration in the plasma up to 48 h after administration. Twenty-four hours after injection, about 22.8% and 25.2% of the injected dose could still be detected in U-SS-M and U-M, respectively. The U-SS-M and U-M preparations could significantly prolong the blood circulation half-life of UA from 1 h to 4.9 and 5.2 h, respectively. Moreover, the blood circulation time between U-SS-M and U-M showed no significant differences.

The prolonged circulation in the blood could promote the accumulation of the nanoparticles in tumor tissues through the EPR effect (Yang et al., 2018). The long half-life of both polymeric micellar preparations contributed to promoting drug accumulation in tumor tissue, and as confirmation, biodistribution assays of all UA formulations were performed using MG-63 tumor-bearing mice. The percentage of the injected dose (%ID) per gram of organ or tissue was used to express the results. As shown in Figure 6(B,C), 6 h and 12 h after the injection, UA mainly accumulated in the liver and kidney was then rapidly redistributed to all the major organs. The U-SS-M and U-M preparations showed a similar biodistribution pattern and mainly accumulated in the liver and kidney and were then cleared by these organs. In the tumor tissue, the concentration of free UA gradually decreased over time, but the amount of UA delivered by the polymeric prodrug micelles gradually increased. The concentration of U-SS-M and U-M in tumor tissue was significantly higher than that of free UA at both 6 h and 12 h after administration. Interestingly, the accumulation of U-SS-M and U-M in tumor tissue was not remarkably different.

### 3.7. *In vivo* antitumor efficiency

Encouraged by the prolonged circulation in the blood and good tumor accumulation of UA polymeric prodrug micelles, the *in vivo* antitumor efficacy of all the UA formulations was evaluated using MG-63-tumor-bearing mice as the animal model and saline-treated mice as the control group. The tumor volume was measured within the treatment period, and the tumor was extracted, imaged, and weighed to calculate the TGI. As shown in Figure 6(D,E), in comparison with the saline group, the tumor growth was only moderately suppressed after treatment with free UA, for which the TGI was 43.5%. In comparison with free UA, the U-M showed stronger tumor inhibition when the TGI was 61.1%, which may be due to the large accumulation of U-M in the tumor tissue due to the EPR effect. As expected, the U-SS-M formulation possessed the strongest tumor inhibitory activity and its TGI was as high as 82.3%, which was significantly stronger than UA and U-M (*p* < .001). The superior tumor suppression of U-SS-M was mainly attributed to its efficient accumulation in the tumor tissue based on the EPR effect, which allowed internalization by cancer cells and rapid and complete drug release. Moreover, because the cellular uptake process, pharmacokinetics, and tumor tissue accumulation of U-M showed no differences when compared with U-SS-M, the superior tumor suppression effect of U-SS-M might be attributed solely to the more efficient intracellular sufficient drug release.

In addition, changes in body weight were monitored to assess the systemic toxicity of UA formulations. As indicated in Figure 6(F), no remarkable body weight loss was observed across all drug treatment groups during the therapy period, indicating that all the UA formulations were safe at these doses.

## 4. Conclusion

In summary, a simple redox-sensitive UA polymeric prodrug was synthesized, able to self-assemble into micelles (U-SS-M) in an aqueous solution. The U-SS-M preparation notably increased the water solubility of UA, significantly prolonged the blood circulation time, and efficiently accumulated in tumor tissue, where it was internalized by tumor cells to allow rapid release of UA in cancer cells in high redox conditions and a weakly acidic environment. Given these advantageous properties, the U-SS-M preparation effectively inhibited OS tumor properties *in vitro* and *in vivo* when compared with control groups. Thus, U-SS-M preparation has great potential to achieve better therapeutic effects in OS patients.
